# Effect of the time interval between denudation and ICSI on fresh embryo transfer reproductive outcomes: a retrospective study

**DOI:** 10.3389/fendo.2024.1283032

**Published:** 2024-02-22

**Authors:** Li Xiao, Luqi Xue, Ju Zhang, Wei Fan, Huili Zhu, Wei Huang

**Affiliations:** ^1^ Department of Obstetrics and Gynecology, West China Second University Hospital of Sichuan University, Chengdu, Sichuan, China; ^2^ Key Laboratory of Birth Defects and Related Diseases of Women and Children (Sichuan University), Ministry of Education, Chengdu, Sichuan, China; ^3^ Department of Obstetrics and Gynecology West China Xiamen Hospital of Sichuan University, Xiamen, Fujian, China

**Keywords:** clinical pregnancy rate, ICSI, oocyte denudation, fresh embryo transfer, time interval

## Abstract

**Purpose:**

This study aims to determine if the incubation after oocyte denudation before Intra-cytoplasmic sperm injection (ICSI) affects the clinical pregnancy rate.

**Methods:**

This was a retrospective, consecutive data analysis of 1370 patients who underwent ICSI cycles at the Department of Reproductive Medicine of West China Second University of Sichuan University (Chengdu, Sichuan) between January 2020 and July 2022. The primary outcome was the clinical pregnancy rate. The second outcome included fertilization rate, biochemical pregnancy rate, and miscarriage rates.

**Results:**

A total of 1370 continuous fresh transferred ICSI cycles were analyzed. Multivariate linear regression and logistic regression analysis of factors related to clinical pregnancy rates revealed that clinical pregnancy rates were significantly associated with denudation (DEN)-ICSI time interval. Long DEN-ICSI intervals are associated with a higher clinical pregnancy rate during fresh embryo transfer.

**Conclusion:**

The DEN-ICSI time interval is an independent factor for clinical outcomes in fresh ICSI transfer cycles.

## Introduction

Intracytoplasmic sperm injection (ICSI) was initially reported in 1992 (1) and represents one of the main breakthroughs in assisted reproductive technology (ART) for fertilization in couples suffering from severe impairment of semen parameters or previous *in vitro* fertilization(IVF) failure. The utilization of ICSI technology in clinics has led to the achievement of numerous healthy live births, resulting in its increasing global application. Throughout the implementation of ICSI technology, denudation (DEN) of cumulus cells is required after oocyte collection. To optimize outcomes, the operation and its appropriate timing have been investigated (2–4).

ICSI should be conducted for a period of time during denudation, as oocytes should be restored during this timeframe (3). In terms of laboratory workflow, researchers have found that performing ICSI and oocyte denudation at any point within the currently accepted timeframe does not appear to negatively impact fertilization and blastulation success in patients with a favorable prognosis (5). It is highly unlikely that variability in the timing of ICSI during this period will have an significant impact on cycle outcome as oocyte appears to possess a physiological tolerance for fertilization during the commonly accepted 2-6 hour window (6).

Nuclear and cytoplasmatic maturations are essential for oocyte competence (7). During follicular development, cumulus cells surrounding the oocyte may promote cytoplasm/nucleus maturation through an autocrine/paracrine mechanism (8). Cumulus cell denudation, the process of removing these cells from the oocyte, may result in damage to the oocyte’s cell membrane due to both the mechanical stress from the pipette and the chemical action of hyaluronidase. The majority of previous studies concluded that OPU-DEN has no significant impact on implantation and pregnancy rates. One study observed that a 1.5-2 hour incubation period prior to DEN resulted in the highest implantation rate, while simultaneously optimizing the pregnancy and live birth rate (9). Moreover, several studies have demonstrated that extending the incubation period between oocyte retrieval (OPU) and DEN can also improve fertilization and blastocyst formation rates, which is conducive to embryo development (2, 9, 10), which recommended that oocytes be incubated for several hours prior to DEN. Alternatively, recent studies conducted in mice have shown that an extended incubation period prior to DEN can lead to oocyte apoptosis, indicating the need for immediate DEN after oocyte retrieval (11).

After denudation, Catherme et al. found a negative correlation between fertilization rate and the interval between DEN and ICSI. The fertilization rate gradually decreased as the time between denudation and ICSI was extended; a longer DEN-ICSI time may lead to adverse pregnancy outcomes (3, 9). However, the impact on pregnancy outcomes was barely evaluated in these studies. Correspondingly, oocytes may undergo cytoplasmic maturation due to incubation prior to ICSI, which would enable oocytes to reach their full activation and development potential and potentially boost the rate of fertilization and pregnancy. In this scenario, it was recommended to carry out the sperm injection immediately after denudation. However, whether there is an optimal implementation time node for these crucial steps is still inconclusive.

Because oocytes require some time to reach full maturity to fully utilize the somatic cell compartment. While time intervals for DEN and ICSI may vary between IVF centers and patients, the optimal timing of oocyte denudation and ICSI remains compromised. Numerous studies from various reproductive centers around the world have been published to investigate the impact of the aforementioned operation nodes on embryo acquisition and pregnancy rate. Conclusions are inconsistent due to a variety of research methodologies and outcome indicators. Therefore, we retrospectively investigated 1370 cases of ICSI in the reproductive medicine department of West China Second Hospital of Sichuan University to optimize the operation time of ICSI and evaluate the relationship between *in vitro* timing intervals and cycle outcome.

## Materials and methods

### Study population and ethical approval

In this retrospective study, data were reviewed from continuous women who underwent ICSI treatment cycles with fresh embryos transferred using partner or donor sperm at the department of reproductive medicine of West China Second University Hospital of Sichuan University between January 2020 and July 2022. Females over 40 years of age were excluded from the study, considering due to the high fertilization failure and poor outcomes. All couples included in the study were not involved in any type of prospective interventional trials. This study conformed to the Declaration of Helsinki for Medical Research involving Human Subjects. Each patient was authorized to receive their clinical data for analysis prior to the start of ICSI treatment. The study was approved by the Ethics Committee of West China Second University Hospital.

### Ovarian stimulation and embryo culture

All patients in our research underwent controlled ovarian hyperstimulation (COH). Even ovarian stimulation protocols in our reproduction medicine center contain GnRH agonist long protocol, GnRH agonist short protocol, and GnRH antagonist protocol, as described elsewhere. The ovarian stimulation protocols and the daily dose of FSH injection were performed according to female ages, ovarian reserve, and various reactions to ovarian stimulation in previous cycles. Patients were monitored by serial ultrasonography until at least two leading follicles reached 18 mm in diameter. Oocyte maturation was accomplished with either human chorionic gonadotropin (hCG; Lizhu Pharmaceutical Trading, China). For patients at high risk for ovarian hyperstimulation syndrome (OHSS), hCG, in combination with leuprolide acetate, was used to trigger ovulation. Oocyte pick up (OPU) transvaginal was accomplished 36-38 h after triggering ovulation.

All the procedures of ICSI were performed according to our routine clinical management. The diversity in the time intervals between different steps of the ICSI cycle was due to the difference in the daily workload of our department. After oocyte retrieval, all oocytes were denuded by brief exposure (60s) to hyaluronidase solution (Irvine Scientific, Santa Ana, CA). ICSI was performed using an Olympus IX73 microscope (Olympus Corporation, Shinjuku City, Japan) with a micromanipulator (Narishige MTK-1, Tokyo, Japan). Additionally, embryos were transferred on Day 3 or Day 5. All transferred embryos were at least four cells with even blastomeres and < 40% fragmentation. All patients were given luteal phase support via the intramuscular injection of progesterone (60 mg/day) or vaginal progesterone gel (90 mg/day) plus oral dydrogesterone (20 mg/day). Two weeks after ET, the pregnancy was assessed by serum β-hCG levels and confirmed by transvaginal ultrasound 4 weeks after ET. Serum β-hCG levels > 50 IU/L were regarded as biochemical pregnancy and the presence of the gestational sac was regarded as clinical pregnancy.

### Time recording

There is no intervention applied in the ICSI cycles. Accurate time intervals between hCG-OPU, OPU-DEN, and DEN-ICSI were recorded by the operator. All the procedures were performed according to our routine clinical management. The diversity in the time intervals between different steps of ICSI cycles was due to the difference in the daily workload of our department. The detailed time was analyzed to evaluate the correlation between time intervals and ICSI outcomes.

### Outcome measures

We regarded the clinical pregnancy rate as the major outcome of our research, while biochemical pregnancy, fertilization, and available embryo rates were secondary outcomes.

### Statistical analysis

SPSS 25.0 (IBM, Armonk, NY, USA) was used to analyze the study data, and GraphPad Prism version 6 (GraphPad Software, La Jolla, CA, USA) was used for graph preparation.

Categorical variables are presented as percentages with 95% CI. Chi-squared or Fisher’s exact tests were used to assess statistically significant differences. Continuous variables are presented as mean ± SD and range. Shapiro–Wilk tests were conducted to investigate whether the data followed a normal (Gaussian) distribution. Kruskal–Wallis or Mann–Whitney U tests were performed to assess statistically significant differences. The P-value of < 0.05 was considered statistically significant. The basic demographic characteristics and main outcome measures were compared among the various time intervals using linear or logistic generalized estimating equations regression. adding simultaneously not only the OPU-denudation and OPU-ICSI time intervals but also the following potentially confounding variables to all regression models: female and male age, duration of infertility, infertility type, AMH, female BMI, number of COCs retrieved, hCG, OPU, DEN, and ICSI time intervals.

## Results

### Baseline characteristics and cycle outcome

This study included a total of 1370 ICSI cycles with fresh embryos transferred between January 2020 and July 2022. **Table 1** reveals the characteristics and reproductive outcomes of included cases. The mean age of females in this study was 31.42 ± 4.14 years (range 20-40 years) and 33.53 ± 5.56 years (range 22-61 years) for males. The mean duration of infertility was 3.75 ± 2.95 years. The percentage of primary infertility in females is 63.94% (876/1370). The percentage of primary infertility in males is 74.31% (1018/1370). The main cause of infertility is severe male infertility factors and previous IVF failure, combined with some female infertility factors such as tubal infertility. The indication of ICSI includes severe oligozoospermia/asthenozoospermia/oligozoospermia, azoospermia, previous IVF failure, immotile sperm and other factors such as semen procurement difficulty. The mean BMI of women was 21.95 ± 2.94. The mean number of oocytes retrieved was 8.01 ± 3.97. The average number of MII oocytes was 6.14 ± 3.28. The mean time in hours for HCG-OPU was 36.36 ± 1.18, 4.29 ± 0.95 for OPU-ICSI, 2.24 ± 0.96 for OPU-DEN, and 2.05 ± 0.96 for DEN-ICSI. The fertilization and clinical pregnancy rates were 85.37% and 51.2%, respectively.

**Table 1:** Baseline characteristics and reproductive outcomes of ICSI cycles.

Parameter	Value
No. of cycles	1370
Female Age (Y)	31.42±4.14
Male Age (Y)	33.53±5.56
Duration of Infertility (Y)	3.75±2.95
Female Infertility Type	
Primary infertility	876
Second infertility	445
Other	49
Male Infertility Type	
Primary infertility	1018
Second infertility	352
AMH	2.91±2.26
BMI	21.95±2.94
Total Gn doses	2502.09±745.55
Days of Gn	10.07±1.75
LH of trigger day	2.00±1.98
E2 of trigger day	2072.26±1044.22
P of trigger day	0.77±0.28
Endometrial thickness of trigger day	5.41±1.10
No. of Oocytes retrieved	8.01±3.97
No. of MII	6.14±3.28
Fertilization rate (%)	85.37±17.86
Blastocyst formation rate(%)	62.40±34.84
Good quality embryo rate (%)	52.33±34.35
HCG-OPU interval (h)	36.36±1.18
OPU-ICSI interval (h)	4.29±0.95
OPU-DEN interval (h)	2.24±0.96
DEN-ICSI interval (h)	2.05±0.96
Type of embryos transferred	
D3	1326
Blastocysts	44
No. of embryos transferred	1.22±0.76
No. of good quality embryos transferred	1.17±0.30
Chemical pregnancy rate (%)	56.8(778/1370)
Clinical pregnancy rate (%)	51.2(701/1370)

Data are presented as mean ± SD or n (%), BMI, body mass index; OPU, oocyte pick-up; DEN, denudation; ICSI, intracytoplasmic sperm injection.

### The relationship between hCG trigger, OPU, denudation, and ICSI timing

Overall, the correlation analysis of the interval times of hCG, OPU, DEN, and ICSI associated with reproduction rate (**Table 2**) showed that DEN-ICSI was significantly correlated with clinical pregnancy rate. Conversely, there was no significant correlation between those interval times with the fertilization rates. A subgroup analysis of the study group compared patients according to female age (≤35 years old and >35 years old). The biochemical pregnancy rates between the two subgroups were similar (57.7% (654/1133) vs. 52.3% (124/237), p=0.131), as was the clinical pregnancy rate per cycle (52.1% (590/1133) vs. 46.8% (111/237), p=0.153). The correlation analysis of the subgroup showed that the DEN-ICSI interval was only significantly correlated with the clinical pregnancy rate in women under 35 years old (**Table 3**).

**Table 2:** Correlation analysis of factors related to the fertilization rate and clinical pregnancy in ICSI cycles.

Time interval	Fertilization rate		Clinical pregnancy	
	Pearson C.C.	P value	Pearson C.C.	P value
HCG-OPU	0.003	0.921	0.026	0.331
OPU-ICSI	0.022	0.420	0.036	0.179
OPU-DEN	0.014	0.601	-0.039	0.151
DEN-ICSI	0.007	0.785	0.070	0.010

OPU, oocyte pick-up; DEN, denudation; ICSI, intracytoplasmic sperm injection.

Pearson C.C. Pearson correlation coefficients.

**Table 3:** Correlation analysis of Time intervals and clinical pregnancy of subgroups.

Time interval	≤35 years (n=1133)		>35 years (n=237)	
	Pearson C.C.	P value	Pearson C.C.	P value
HCG-OPU	0.016	0.592	0.056	0.394
OPU-ICSI	-0.045	0.127	0.078	0.232
OPU-DEN	0.033	0.268	0.055	0.396
DEN-ICSI	-0.079	0.008	0.024	0.708

OPU, oocyte pick-up; DEN, denudation; ICSI, intracytoplasmic sperm injection.

Pearson C.C. Pearson correlation coefficients.

**Figure 1:**
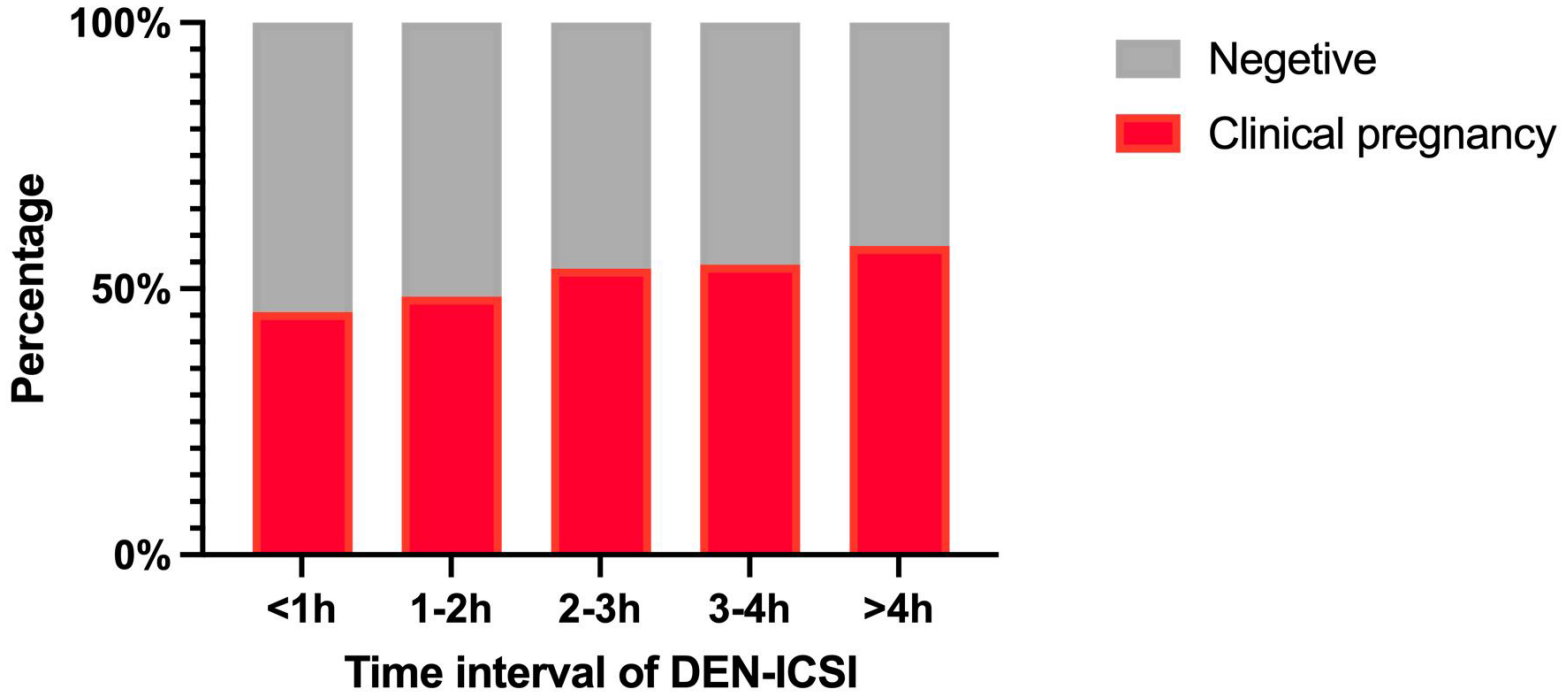
Clinical pregnancy rate according to the time interval of DEN-ICSI Note: DEN denudation, ICSI intracytoplasmic sperm injection.

To assess in more detail the relationship between denudation and injection, a final analysis was additionally performed for the main clinical outcome, namely the possibility of an interaction between these time intervals. To evaluate this, a multivariable analysis was conducted to adjust outcome measures for confounding variables (age, duration of infertility, infertility type, AMH, BMI, hCG, OPU, DEN and ICSI time intervals, type of transferred embryos, and number of embryos transferred and so on), which is shown in **Table 4**. Clinical pregnancy rates were only significantly related to AMH, type of transferred embryo, number of good-quality embryos transferred, and DEN-ICSI time interval. To obtain a comprehensive correlation, the DEN-ICSI time interval was separated into distinct segments: 0-1, 1-2, 2-3, 3-4, and > 4 h. **Figure 1** shows the overall association between the DEN-ICSI interval and clinical pregnancy rate. There was an increase in the clinical pregnancy rate with longer DEN-ICSI intervals.

**Table 4:** Multiple regression analysis of potential factors associated with clinical pregnancy.

Logistic regression	Variable	OR	P-value
Clinical pregnancy	AMH	1.056	0.033
	DEN-ICSI time interval	1.123	0.049
	Type of embryos transferred	2.532	0.005
	No. of good quality embryos transferred	1.884	0.000

OPU, oocyte pick-up; DEN, denudation; ICSI, intracytoplasmic sperm injection.

## Discussion

The findings of the present study demonstrate that the DEN-ICSI time interval serves as an independent predictive factor of clinical outcomes in ICSI cycles. Consistent with previous research, most researchers have identified a correlation between the DEN-ICSI interval and fertilization rate. However, few studies address the impact of a raised DEN-ICSI interval on clinical pregnancy outcomes. This study elucidates that optimizing the clinical pregnancy rate in ICSI cycles can be achieved by performing ICSI within 4 hours of oocyte denudation. Furthermore, Extending the *in vitro* oocyte culture time following denudation and before ICSI is independently associated with an increase in clinical pregnancy.

It has been confirmed that while oocyte spindles are visible upon observation, most of which appear 39-40.5 hours after hCG administration, fertilization, and implantation rates are higher, but after this time, the oocyte begins to deteriorate. Therefore, ICSI should be performed 39-40.5 hours after hCG administration (12). In our study, the OPU-ICSI time interval is also restricted to this requirement. Unlike hCG-OPU time intervals, the time in which oocytes were cultured *in vitro* prior to ICSI had no impact on reproductive outcome. Consistent with a previous large retrospective study finding that oocyte culture duration did not affect ICSI outcome since it did not compensate for insufficient post-priming exposure to the follicular environment (13), our findings show that there was no difference in either fertilization rate or clinical pregnancy rate with the extension of OPU-DEN time interval.

Although numerous studies have attempted to determine the optimal time for cumulus cell removal in ICSI procedures, no consensus has been reached. Vande et al. (14)found that culturing oocytes with CCs prior to ICSI had no effect. Therefore, they hypothesized that if synchronization of nuclear and cytoplasmic maturation occurs at the time of OPU, the surrounding cells have no effect. Our results indicate that the DEN-ICSI time interval acted as an independent predictor of clinical outcomes in ICSI cycles, which is partially consistent with a previous large retrospective study (4). However, more than 4 hours of time interval showed no decrease in clinical pregnancy rate in our study compared to their result, which showed a significant decrease in clinical pregnancy rate when the interval was more than 4 hours. They excluded patients with few oocytes retrieved (≤4) and a low fertilization rate (≤25%), which may contribute to this difference. Our results may be more suggestive for general patients with ICSI cycles. The present study revealed that the fertilization rate did not change belonging to the DEN-ICSI interval lengthened. This result was consistent with previous results (3, 4), which both found that the time before ICSI had a significant positive impact on fertilization. Moreover, Rienzi et al. showed that the optimal time range for better fertilization and embryo development rates in ICSI cycles appeared to be between 3 and 12 h after oocyte retrieval when oocytes tended to reach cytoplasmic maturation (15). Recent reports on murine COCs culture suggest that apoptosis increases in oocytes with intact cumulus cells after long-term culture (11, 16). Oocyte denudation should follow oocyte retrieval to ensure optimal success.

Numerous studies have attempted to establish an optimal time to perform ICSI after DEN, but no consensus has been reached. In 1998, Van et al. revealed for the first time that the interval between OPU and ICSI was irrelevant to ICSI outcomes and that MII oocytes may not require additional cytoplasmic maturation (17). Since the prolonged culture of oocytes with intact cumulus cells induces apoptotic changes, it has been recommended that oocyte denudation be performed as soon as possible after oocyte retrieval (11). Hyaluronidase treatment is commonly used to eliminate cumulus cells from COCs and is a vital component of ART, like ICSI. Treatment of human oocytes with hyaluronidase has been shown to increase the rate of parthenogenetic development and have a negative effect on fertilization rates (18–20). Further research have demonstrated that hyaluronidase may cause an increase in Ca^2+^ levels in oocytes due to a change in the function of Ca^2+^ channels in the cell membrane, which increased Ca^2+^ levels in the oocytes and led to a reduction in embryonic development (18). It has been reported that an increase in Ca2+ levels in the physiological range triggers the generation of nitric oxide (NO), reduction of maturation promoting factor (MPF) level, and meiotic resumption from diplotene arrest in rat oocyte (21). In contrast, Catherme Patrat et al. analyzed 110 ICSI cycles by logistic regression and determined that the optimal time to perform DEN was within three hours of OPU in terms of fertilization rate (9). Differences in results between previous and current studies may be due to different laboratory routines, inclusion and exclusion criteria, number of populations, and the observation outcomes. In contrast to their reports, which included only laboratory data, our results focus more on pregnancy outcomes, one of the best indicators of embryo quality and patient needs.

As a limitation, the mean blastocyst rate(m-BR) was not considered as one of the main outcome index in the present study. A prospective randomized study examined the impact of intact cumulus cell removal on fertilization and embryonic development, showing that the percentage of good-quality blastocysts was significantly higher with shorter incubation after denudation (2). We will further investigate all consecutive ICSI with or without fresh transfers, particularly in Preimplantation Genetic Testing (PGT) patients. Our study’s strengths include its large sample size, its analysis of fresh transfer results and fertilization rates, and examination of DEN-ICSI time intervals. This highlights the fact that the main limitation of the study is that it was conducted in retrospect, which raises the possibility of important unaccounted-for confounders. Therefore, further investigation is needed as to whether ICSI should be performed after a period of recovery following degranulation. To optimize the exact timing of the ICSI procedure, it is necessary to conduct additional multicenter, randomized, controlled studies with large sample sizes in the future.

## Conclusions

Based on our results, our study provides evidence that incubation time after denudation had a positive effect on pregnancy outcomes for ICSI. At least two hours of incubation after oocyte denudation contribute to reproductive results in ICSI cycles. This study not only provides new insights into the definition of oocyte quality and *in vitro* aging in human cells, but also provides simple but potentially relevant measures in routine laboratory procedures. It should be further investigated and consolidated through prospective, randomized, controlled trials.

## Data availability statement

The raw data supporting the conclusions of this article will be made available by the authors, without undue reservation.

## Ethics statement

The studies involving humans were approved by the Ethics Committee of West China Second University Hospital. The studies were conducted in accordance with the local legislation and institutional requirements. The participants provided their written informed consent to participate in this study. Written informed consent was obtained from the individual(s) for the publication of any potentially identifiable images or data included in this article.

## Author contributions

LX: Data curation, Formal analysis, Methodology, Software, Writing – original draft, Writing – review & editing. LX: Data curation, Formal analysis, Methodology, Writing – original draft, Writing – review & editing. JZ: Data curation, Methodology, Writing – review & editing. WF: Conceptualization, Supervision, Writing – review & editing. HZ: Conceptualization, Methodology, Writing – review & editing. WH: Conceptualization, Investigation, Project administration, Supervision, Writing – review & editing.
